# Thermal physiology of the lactating nipple influences the removal of human milk

**DOI:** 10.1038/s41598-019-48358-z

**Published:** 2019-08-14

**Authors:** Hazel Gardner, Ching Tat Lai, Leigh C. Ward, Donna T. Geddes

**Affiliations:** 1 School of Molecular Sciences, Faculty of Science, University of Western Australia, Perth, Australia; 2 School of Chemistry and Molecular Biosciences, University of Queensland, Brisbane, Queensland Australia

**Keywords:** Physiology, Endocrinology

## Abstract

The nipple has a critical role in successful breastfeeding. Nipple trauma or pain may negatively impact breastfeeding duration which has significant public health implications. The aim of this study was to examine changes in nipple temperature during breastfeeding and pumping within participants. Thirty lactating women participated in two pumping (electric breast pump) and one breastfeeding session. Nipple temperature of both breasts was monitored for two minutes before and after each session with the non-pumped/non-suckled nipple temperature recorded throughout each session. The mean increase in nipple temperature after milk removal by the infant was 1.0 ± 1.6 °C (range −3.2–3.2) and after expression was 1.8 ± 1.4 °C (range −0.9–6.1). Nipple temperature pre expression was significantly lower than post expression (Pre 32.6 ± 1.6, Post 34.3 ± 1.3, p < 0.001) with no difference between the two pumping sessions. For every 1 °C rise in temperature an additional 10 mL of milk was removed on average. The breastfed nipple temperature was significantly lower pre feed than post feed (Pre 32.4 ± 1.6, Post 33.2 ± 1.2 p = 0.01) with a significant but smaller change in nipple temperaturecompared to pumping (Breastfeed 1.0 ± 1.6, Pumping 1.7 ± 1.4, p = 0.03). Nipple temperature increases during pumping and breastfeeding suggesting the breasts have a similar physiological response to different stimuli. Further, the increased temperature potentially plays a role in effective milk removal.

## Introduction

The mammary gland undergoes complex changes to reach its functional capacity during pregnancy and lactation. Despite the nipple being integral to the release of breast milk for the infant while feeding, and also stemming leakage between breastfeeds, little is known about its anatomy and function during lactation.

The structure of the mammary gland includes intertwined and overlapping ductal systems which are anatomically distinct^1^. Orifices from these ductal systems open onto the nipple, which is usually more pigmented than the surrounding skin and becomes darker during pregnancy and lactation^2,3^. The dermal surface of the nipple is covered with epidermal ridges, which may have a role in protecting the nipple from trauma during sucking^4,5^. The end of the nipple has sebaceous glands, which open onto the tip of the nipple as well as within the ducts, and may play a role in protecting the nipple from cracking^5,6^. The nipple has abundant smooth muscle fibres that are arranged in a radial (muscle of Myerholz) and circular fashion (muscle of Sappey) forming a mesh like structure in the connective tissue around the ducts at the tip of the nipple, and larger fibres that are found along the ducts extending into the nipple. Contraction of the smooth muscle is moderated through the sympathetic adrenergic nerves and enhances nipple erection, which facilitates breastfeeding^7–10^.

Increased blood flow may influence the skin temperature of the mammary gland. Breast skin temperature is known to increase by one degree celsius during pregnancy, from 33.3 ± 0.1 °C to 34.2 ± 0.1 °C and a further one degree celsius increase during lactation as measured by Burd *et al*.^11^ one day postpartum. The areola is typically warmer compared to the rest of the breast tissue (34.6 ± 1.4 °C vs 34.0 ± 2.0 °C), possibly as a signal or stimulation for the infant to commence suckling or to indicate the location of the nipple^12,13^. In contrast, the nipple is significantly cooler than the areola (33.7 ± 1.4 °C and 34.8 ± 1.1 °C respectively) which may help constrict the nipple to prevent milk leakage between breastfeeds. Nipple temperature has been shown to increase during pumping studies (increased by 0.6–1.8 °C)^14,15^ suggesting that there is increased blood flow to the nipple, supported by studies in lactating women showing increased breast skin temperature (0.4 to 1 °C)^16^ and mammary blood flow across a breastfeed (BF)^17^. These changes are also evident in the rat model^18^. This phenomenon could be in part due to the release of oxytocin (OT) causing vasodalition of the venous superficial plexus of the breast resulting in increased skin temperature. There is an assumption that the same changes in temperature and mammary blood flow would be evident in the nipple. However vasoactive peptides released from local nerves serve to regulate blood flow; neuropeptide Y (NPY) which is a vasoconstrictor and calcitonin gene-related peptide (CGRP) a vasodilator may also play a role particularly in the nipple during pumping or breastfeeding^18^.

The internal thoracic artery supplies the majority of blood to the nipple via the first to fourth intercostal branches^9^. Oxytocin not only stimulates milk ejection but also increases blood flow to the breast via vasodilation^17,19^. Oxytocin release in response to nipple stimulation is systemic, and should therefore also impact nipple temperature on the opposite breast, but this response has not yet been documented.

Thermal imaging has been widely used in the dairy industry to measure changes in teat temperature during milking^20,21^. In women, thermal imaging has been used to examine changes in temperature associated with pumping. The temperature of the nipple increased by approximately 0.6 °C after 5 minutes of pumping and 1.8 °C after 15 minutes of pumping using a breast shield at ambient temperature but returned to baseline levels within two minutes of cessation of pumping^14,15^. The warming effect of the infants mouth has been replicated during pumping through the use of warmed shields (39 °C). This resulted in more expedient removal of 80% of the available milk when combined with the participants maximum comfortable vacuum. This effect was attributed to the warmth relaxing the nipple allowing the nipple ducts and nipple pores at the nipple tip to expand thus facilitating the removal of milk. In contrast, when cold ultrasound gel was applied to the nipple, nipple ductdiameters were reduced suggesting that pumping with kits that have been stored in the refrigerator may not be conducive to efficient milk removal^14^.

The nipple is a critical factor in successful milk removal from the breast by the infant, and hence better understanding of nipple physiology will allow identification of changes associated with suboptimal breastfeeding and pumping, and would facilitate targeted management strategies to improve lactation outcomes for the mother and infant. Nipple pain or anomalies have been implicated as major factors in the cessation of breastfeeding^22,23^, yet examination of the nipple before and after breastfeeding or pumping has not been carried out. Indeed, extreme changes in nipple temperature may signify reduced blood flow due to poor fitting breast shields, extended duration of pumping, or perhaps infection.

The aim of this study was to use thermal imaging to measure temperature changes of both nipples during breastfeeding and milk expression using an electric breast pump.

## Results

Participant characteristics are presented in Table 1. All participants had normal milk productions within the range reported by Kent *et al*.^24^. There was no significant difference in ambient temperature between the sessions (S) (S1: 22.4 ± 0.4 °C, S2: 22.1 ± 0.9 °C, S3: 22.1 ± 0.5 °C). No relationship was observed between the volumes of milk removed and the ambient temperature (S1 p = 0.32, 95% C.I.: 67 to 101.5 ml, S2 p = 0.13, 95% C.I.: 52.4 to 86.9 ml, S3 p = 0.87, 95% C.I.: 47.4 to 81.9).

**Table 1:** Participant characteristics.

		Mean (SD)	Range
Mother	Age (years)	31 (3)	21–38
	Parity	2 (1)	1–5
	Body Mass Index	28 (6)	19–54
	Milk production (ml/24 h)	778 (169)	484–1155
Infant	(female n = 13, male n = 17) Gestational age (weeks)	39 (1)	37–41
	Birthweight (g)	3653 (435)	2660–4645
	Current age (weeks)	18 (7)	5–33

### Nipple temperature changes during pumping and breastfeeding

**Figure 1:**
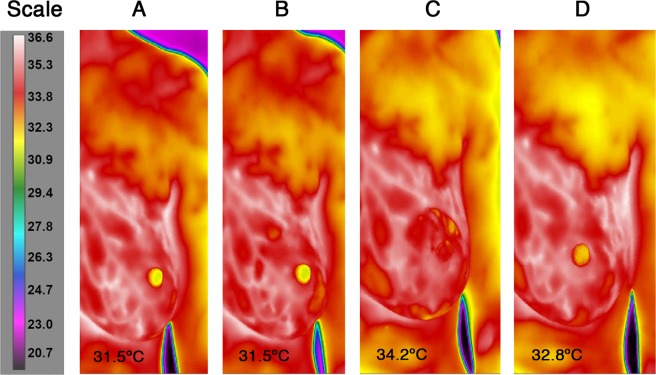
Nipple temperature before and after a pumping session. (**A**) 2 minutes prior to pumping, (**B**) immediately before pump was started, (**C**) immediately after pump stopped, (**D**) 2 minutes after 10 minutes of pumping.

The nipple temperature increased significantly during both the pumping and breastfeeding sessions (pumping, mean difference: 1.73 °C, p < 0.001, 95% C.I.:1.36 to 2.10; breastfeed p = 0.01, mean difference: 0.95 °C 95% C.I.: 0.36 to 1.55 Fig. 1- shows changes occurring on a pumped breast). Further, the magnitude of change was not different between the left and right nipple (Table 2).

**Table 2:** Nipple temperatures of the left and right nipples on the breast that was pumped (2 pumping sessions on alternate breasts).

	Pump 1 (n = 30)		Pump 2 (n = 30)		p
	Mean SD	Range	Mean SD	Range	
Volume (ml)	83 ± 55	4–264	70 ± 51	14–276	0.06
Nipple temperature before (°C)	32.5 ± 1.5	29.6–35.1	32.7 ± 1.8	28.4–34.9	0.19
Nipple temperature after (°C)	34.1 ± 1.3	30.4–36.9	34.6 ± 1.3	31.2–36.8	0.64
Change in nipple temperature (°C)	1.7 ± 1.3	−0.9–4.3	1.9 ± 1.6	−0.6–6.1	0.37

There was no difference in the degree of change between the two pumping sessions which were conducted on different breasts for all participants (p = 0.37, 95% C.I.: 1.22 to 1.76) (Table 2).

**Figure 2:**
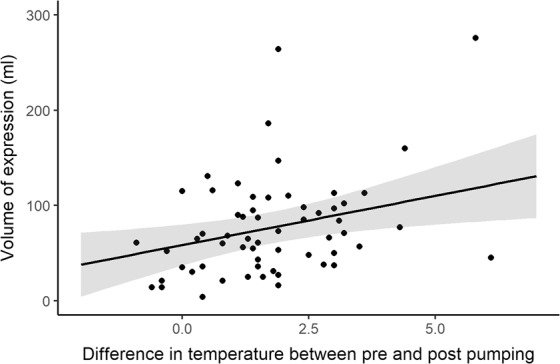
Milk volume removed and temperature change during pumping (n = 60 shaded area represents 95% confidence limits).

The volume of milk removed during the pumping sessions was positively associated with the increase in nipple temperature (p < 0.02, 95% C.I.: 2.4 to 18.2). An increase of 1 °C in nipple temperature was associated with a mean estimate of a 10 ml increase in milk volume expressed (Fig. 2).

Breastfeed duration was dependent on the infant and lasted between 2.2 and 30 minutes (mean 9 mins ± 5). The changes in nipple temperature on the suckled breast before and after breastfeeding were not related to the volume of milk removed by the infant (p = 0.70). The duration of the breastfeed was determined by the infant and was not associated with changes in nipple temperature (p = 0.30).

Table 3 shows the temperature changes when the same breast was pumped and fed from in two different sessions. The nipple temperature post pump and the magnitude of change was much larger on the pumped breast.

**Table 3:** Changes in nipple temperature during pumping and breastfeeding on the same breast.

	Pump 2 (n = 30)		Breastfeed (n = 30)		p
	Mean ± SD	Range	Mean ± SD	Range	
Volume	70 ± 51	14–276	65 ± 36	10–158	0.60
Nipple temperature before (°C)	32.7 ± 1.8	28.4–34.9	32.4 ± 1.4	30.3–35.2	0.16
Nipple temperature after (°C)	34.6 ± 1.3	31.2–36.8	33.2 ± 1.2	31.1–35.4	0.00
Change in nipple temperature (°C)	1.9 ± 1.6	−0.6–6.1	1.0 ± 1.6	−3.2 ± 3.2	0.03

### Nipple temperature changes on the non pumped breast

There were no significant differences in the nipple temperature pre, post or in relation to the magnitude of change between the right and left nipple (Table 4).

**Table 4:** Nipple temperature changes on the non pumped breast.

Temperature	Right Nipple (n = 30)			Left Nipple (n = 30)			p
	Mean	SD	Range	Mean	SD	Range	
Opposite nipple, before (°C)	32.6	1.8	28.5–35.0	32.9	1.4	30.0–35.0	0.51
Opposite nipple, after (°C)	32.0	1.8	28.5–35.0	31.9	1.6	28.4–34.5	0.73
Change (°C)	−0.6	1.0	−3.8–0.6	−1.0	1.0	−3.6–0.7	0.16

**Figure 3:**
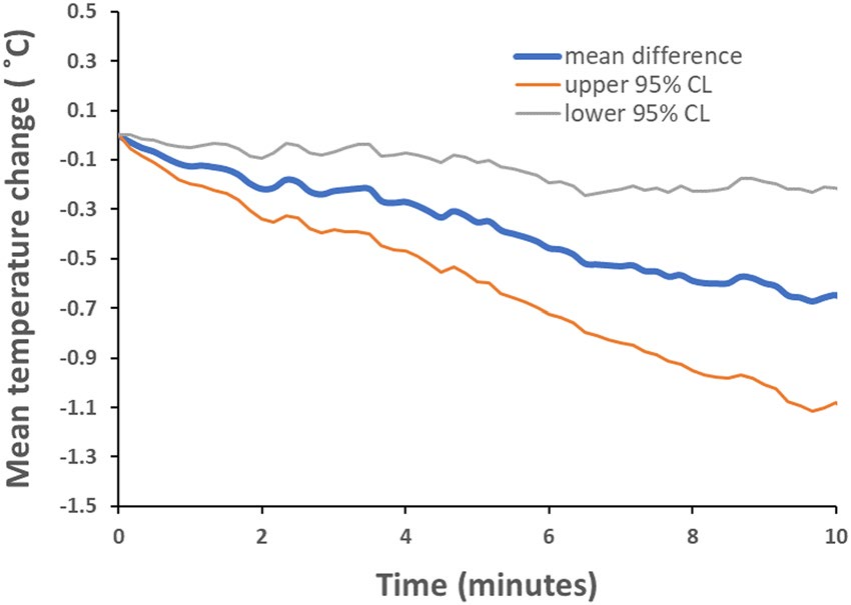
Mean change in nipple temperature on the non-pumped breast during pumping for all sessions (n = 60).

There was a significant difference between the nipple temperature pre and post pumping on the non-pumped breast (pre: 32.7 ± 1.6 °C, post: 31.9 ± 1.7 °C, p < 0.001, Mean difference: −0.8 °C, 95% C.I.: −1.05 to −0.55). The nipple on the opposite breast was monitored over the entire pumping session and temperature decreased gradually over the course of the session (Fig. 3) (data from the other breast during breastfeeding was intermittent therefore is not reported).

**Figure 4:**
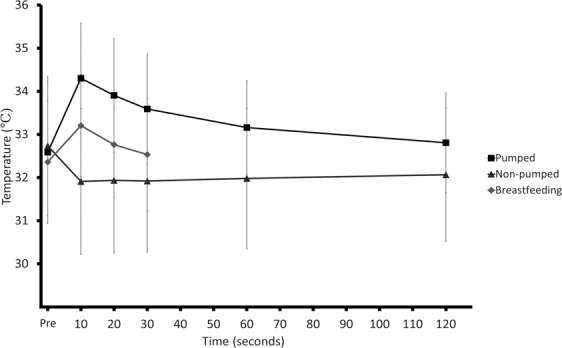
Time taken to return to baseline nipple temperature (Nipple temperature before pumping and breastfeeding and the opposite breast immediately before the pump or infant began to remove milk and at various time intervals after the cessation of pumping or suckling).

By 30 seconds after the end of the breastfeed, no difference between pre and post nipple temperatures remained (p = 0.60). There were no differences between the pre and post measures at 2 minutes after pumping on the pumped breast (p = 0.1) or the non-pumped breast (p = 0.08) (Fig. 4).

## Discussion

This study confirmed that nipple temperature increases significantly during both pumping and breastfeeding on the ipsilateral breast, implying similar heamodynamic changes occurred in response to the different methods of milk removal. No differences in temperature change were noted between right and left nipples suggesting a similar response irrespective of breast. Furthermore, with increasing nipple temperature during pumping more millk was removed from the breast. Thus is appears that changes in nipple temperature and blood circulation are potential mediators of effective milk removal from the breast.

Previously it has been reported that an individual’s left and right breast share the same anatomical features such as breast tissues and number of milk ducts^25^. In addition milk ejection occurs in both breasts simultaneously^26^.

The nipple temperature of the pumped breast increased (Fig. 1) during 92% of milk expression sessions (Table 2) and each 1 °C increase in nipple temperature was associated with an additional 10 ml of milk removed (Fig. 2). Increased temperature is regarded as a proxy for increased blood flow. The increase in nipple temperature could be due to the localised action of vasodilatory neuropeptides e.g. CGRP^16,17,27^. Elevated nipple temperature may also be due, in part, to the warming of the nipple-areolar complex by the shield and warm milk (37 °C) moving through the nipple pores, allowing dilation of the nipple orifices and relaxation of the smooth muscle to facilitate milk removal. Indeed warm breast shields have been shown to remove 80% of total volume pumped more rapidly (4.8 minutes) than ambient temperature shields (6.5 minutes)^14^. Whilst warmth appears conducive to milk removal, it is possible the application of cold breast shields (stored in the refrigerator) would be detrimental to efficient pumping.

Breastfeeding showed a significant increase in nipple temperature consistent with pumping, although the changes were of a smaller magnitude. We did not find a relationship between nipple temperature change and the volume of milk removed by the infant. This absence of an association is likely due to variation in the duration of breastfeeding (BF) compared to pumping (mean 9 ± 5 min, range 2.2 to 30 min) as well as the proportion of nutritive sucking and the regulation of milk removal according to infant appetite. However, warmth applied to the nipple by the infant (35.5–37.5 °C) is similar to the temperature applied by a warm shield during pumping. This study found that warmth resulted in more rapid removal of the volume of milk pumped^14^. Thus temperature may impact milk removal in the breastfeeding infant but may be masked by the inability to standardise conditions between infants. Similarly, it has been shown that infants are able to remove 80% of the milk they require in the first 4 minutes of a feed^28^ and therefore warmth appears to have the greatest effect on the release of milk in early feeding or pumping, which is equivalent to the first two milk ejections^14^.

Surprisingly, the temperature of the non-pumped nipple decreased significantly across a pumping session (Fig. 3). The non-pumped breast was exposed to ambient temperature, and the nipple may be constricted to prevent breast milk leaking from this breast during pumping or breastfeeding, resulting in decreased blood flow and consequently lower temperature. The mechanisms by which nipple blood flow is reduced are not clear; however, locally active vasoconstrictor neuropeptide Y is thought to reduce oxytocin release thereby regulating mammary blood flow and may influence contraction of the nipple via smooth muscle receptors^18,29,30^. Conversely the impact of warmth on the nipple may be far greater than realised, and be necessary for smooth mucle relaxation facilitating elongation of the nipple to facilitate milk flow through the nipple ducts. Further studies are necessary in lactating women to confirm this hypothesis^18^.

The ambient temperature was not found to have an impact on milk removal in this study, which may be due to the warmth applied to the nipple by the infant and the pumping process.

The increased nipple temperatures recorded during breastfeeding returned to baseline levels more rapidly (30 seconds) than pumping (two minutes; Fig. 4), which supports findings from a previous pumping study^15^. The more rapid return temperature to pre breastfeed levels could be due to the smaller increase in temperature (1 °C less) and the shorter duration of breastfeeding compared to pumping. In contrast to the nipple temperature, mammary skin temperature remains high, persisting for at least five minutes. Increased blood flow in the lateral thoracic artery has been reported to take 0.5–1 hour to return to pre-feed levels^17^. Prolonged increase in mammary skin temperature compared to the nipple may be due to the persistent increased superficial vasodilation induced by oxytocin^16^.

Nipple temperature measurement potentially provides a rapid, non-invasive method to investigate common lactation issues associated with the nipple such as vasoconstriction, nipple oedema, nipple trauma and nipple infection. Indeed thermal imaging has been used in dairy cows to show congestion of the teat which is associated with reduced blood flow and reduced milk removal^20,21^. The application may not be limited to anomalies, but also has potential to identify milk ejection and timing of onset of secretory activation^11,17,31^. Our results serve as a reference of normal changes in temperature that occur during pumping and breastfeeding in women with no lactation problems.

## Conclusion

This study demonstrated that nipple temperature increased during pumping sessions, with the extent of this increase being associated with enhanced milk removal. The magnitude of change in nipple temperature after breastfeeding was significantly less than pumping but was not associated with volume of milk consumed by the infant. In addition, left and right nipples respond to the breastfeeding infant or breastpump with a similar change in nipple temperature. Thermal imaging could potentially be a useful tool to monitor a range of conditions in lactating women.

## Materials and Methods

Thirty lactating women were recruited, either through the Australian Breastfeeding Association or by social media. All mothers provided informed consent to take part in the study, which was approved by the University of Western Australia’s Human Research Ethics Committee (RA/4/1/7897). All methods were performed in accordance with the relevant guidelines and regulations. Demographics were collected by completion of a background questionnaire. The majority of sessions (87 out of 90) were undertaken in participants’ homes, with only one participant completing sessions (3 sessions) in the laboratory at the University of Western Australia.

**Figure 5:**
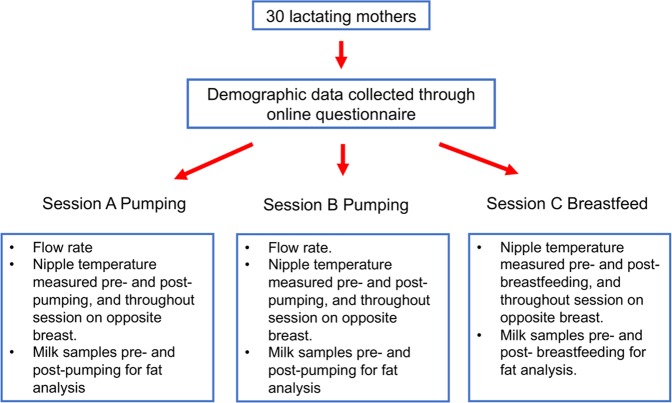
Study design.

Each participant took part in three sessions (n = 90) (Fig. 5). The sessions were conducted one week apart on the same day of the week at the same time of day. Each session was randomised such that each participant pumped once from the left and right breast and the breastfeed was from either breast. Participants pumped for 10 minutes after milk ejection (Symphony, Medela AG, Baar, Switzerland was used for all pumping sessions). Infant milk intake was measured using the test weighing method^32^ and duration of the breastfeeding session was dependent on the infant. As the majority of sessions were conducted in the homes of the participant the baby was always present during the research sessions. Milk samples from study sessions were transported to the laboratory in a cool box filled with ice.

### Milk flow

Milk flow rate was measured using the method described by Prime *et al*.^26^; a continuous balance was used to measure cumulative milk volume and flow rate (g/s).

### Nipple temperature

Nipple temperature data were collected using a FLIR T650sc thermal camera (Flir Systems Inc, Oregon, USA), which has thermal sensitivity <20 mK @ 30 °C (accuracy 0.02 °C), with image capture of 30 frames per second and spectral range 7.5–14 µm.

Ambient temperature was recorded prior to each session and the breasts were exposed to room temperature during the set up period to equilibrate. The temperature was then recorded for two minutes before and after pumping or breastfeeding. At the end of pumping or breastfeeding the nipple was gently dried to remove any milk droplets immediately prior to recording the temperature. The temperature was simultaneously recorded for the opposite nipple throughout the session. For the continuous measurement of the non pumped and non suckled breast the milk flow data and nipple temperature data were synchronised for each session in preparation for analysis. The data were plotted to provide a visual image of the temperature changes occurring on the nipple of the opposite breast.

### Calculation of percent available milk removed (PAMR)

Each participant completed a 24 hour milk profile using the test weighing method^32^. Samples of 1–2 ml were collected in 5 ml polypropylene tubes (P5016SL, Techno Plas Pty Ltd, SA, Australia) before and after each feed or breast expression. These samples were frozen until collected and transported to the laboratory by the research team, where they were analysed for fat content using the creamatocrit method^33^. Participants entered data from each feed or expression electronically, and these data were then used to calculate breast fullness and storage capacity based on the method described by Kent *et al*.^34^. The volume and cream content of milk removed during the experimental sessions were added to this dataset to calculate the available milk, breast fullness and PAMR during each session.

### Data analysis

Nipple temperature data were prepared for analysis using the FLIR software provided with the camera (FLIR Research IR, version 4.30.1.70, USA).

Given this was a pilot study 30 individuals were included. With 30 individuals if the true difference in nipple temperature is 0.6 (and assuming a standard deviation of 0.6), we will be able to reject the null hypothesis that the temperature is the same before and after feeding with probability (power) 0.967. The Type I error probability associated with this test of this null hypothesis is 0.05.

Students T tests were used for descriptive statistics. Linear mixed effect models were used to analyse the difference in nipple temperature and milk volume. Differences in nipple temperature or milk volume were modelled with fixed effects for breast, pumping/breastfeeding session, degree of fullness and initial nipple temperature. In addition, differences in the milk volume as explained by pumping session and room temperature and nipple temperature as explained by pumping session (Fig. 2) were also modelled. The interactions of the fixed effects were also tested in the models. All models included the random effect of participant. Contrasts were examined for all pairwise comparisons with Tukey corrections.

Statistical analyses were carried out using R version 3.4.4^35^ and R Studio Version 1.0.136^36^ using nlme package^37^ for linear mixed effect modelling. Significance was set at the 5% level.

To analyse nipple temperature recordings, a nine pixel cursor was applied to the tip of the nipple and the average temperature was recorded for every 300^th^ frame.

## Data Availability

The datasets generated during and/or analysed during the current study are available from the corresponding author on reasonable request.
